# Identification and Validation of Ferroptosis-Related LncRNA Signatures as a Novel Prognostic Model for Colon Cancer

**DOI:** 10.3389/fimmu.2021.783362

**Published:** 2022-01-26

**Authors:** Zhiwei Wu, Zhixing Lu, Liang Li, Min Ma, Fei Long, Runliu Wu, Lihua Huang, Jing Chou, Kaiyan Yang, Yi Zhang, Xiaorong Li, Gui Hu, Yi Zhang, Changwei Lin

**Affiliations:** ^1^ Department of Gastrointestinal Surgery, The Third XiangYa Hospital of Central South University, Changsha, China; ^2^ School of Life Sciences, Central South University, Changsha, China; ^3^ Department of General Surgery, Affiliated Hospital of Xuzhou Medical University, Xuzhou, China

**Keywords:** lncRNAs, ferroptosis, colorectal cancer, prognostic signature, immune microenvironment

## Abstract

**Background:**

Ferroptosis is a newly defined form of programmed cell death that plays an important role in many cancers. However, ferroptosis-related lncRNAs (FRLs) involved in the regulation of colon cancer are not thoroughly understood. This study aimed to identify a prognostic FRL signature in colon cancer and explore its potential molecular function.

**Methods:**

RNA-seq data and relevant clinical information were obtained from The Cancer Genome Atlas (TCGA) database, and a list of ferroptosis-related genes was extracted from the FerrDb website. Analysis of differentially expressed FRLs was performed using the ‘limma’ package in R software. By implementing coexpression analysis and univariate Cox analysis, we then identified prognostic FRLs. Using Cox regression analysis with the least absolute shrinkage and selection operator (LASSO) algorithm, we constructed a prognostic model based on 4 FRLs. We evaluated the prognostic power of this model using Kaplan–Meier (K-M) survival curve analysis and receiver operating characteristic (ROC) curve analysis. Moreover, the relationships between the signature and immune landscape, somatic mutation and drug sensitivity were explored. Finally, *in vitro* experiments were conducted to validate the functions of AP003555.1 and AC000584.1.

**Results:**

A 4-FRL signature was constructed. Two risk groups were classified based on the risk score calculated by this signature. The signature-based risk score exhibited a more powerful capacity for survival prediction than traditional clinicopathological features in colon patients. Additionally, we observed a significant difference in immune cells, such as CD4+ and CD8+ T cells and macrophages, between the two groups. Moreover, the high-risk group exhibited lower IC50 values for certain chemotherapy drugs, such as cisplatin, docetaxel, bleomycin or axitinib. Finally, the *in vitro* experiments showed that ferroptosis processes were suppressed after AP003555.1 and AC000584.1 knockdown.

**Conclusion:**

The proposed 4-FRL signature is a promising biomarker to predict clinical outcomes and therapeutic responses in colon cancer patients.

## Introduction

Colon cancer is the third most-diagnosed cancer and the second leading cause of cancer-related deaths in the world. Colon cancer seriously endangers human health (1). According to the latest online epidemiological database, there were more than 1.9 million new colon cancer cases in 2020, and 0.9 million deaths were recorded in the same year (2). The incidence rate and mortality rate have continuously risen in recent years. Even with the rapid development of cancer screening methods, many patients are diagnosed at an advanced stage with multiple symptoms, such as haematochezia or colonic obstruction (2). However, there are only a few effective therapeutic targets for colon cancer patients (3). Therefore, along with improvements in surgical treatments and chemoradiotherapies, it is also crucial and important to explore additional diagnostic biomarkers and possible therapeutic targets.

Ferroptosis is a newly defined form of regulated cell death driven by loss of activity of the lipid repair enzyme glutathione peroxidase 4 (GPX4) and the subsequent accumulation of lipid-based reactive oxygen species (ROS), particularly lipid hydroperoxides (4). This type of programmed cell death has been associated with carcinogenesis, intracerebral haemorrhage, degenerative diseases, stroke, and kidney degeneration (5). Ferroptosis has unique morphological and bioenergetic features that can be easily distinguished from other types of programmed cell death, such as apoptosis or necrosis. Currently, inducing cancer ferroptosis is considered a promising therapeutic strategy, especially for drug-resistant cancers (6). However, only a few ferroptosis-related therapeutic targets have been identified in colon cancer (7–9). Thus, further clinical sample-based screenings for ferroptosis-related genes (FRGs) are necessary for colon cancer diagnoses and treatments.

Long noncoding RNA (lncRNA) refers to a type of noncoding RNA more than 200 nucleotides in length. LncRNAs constitute a major class of transcripts that are encoded by the genome but are mostly not translated into proteins (10). In the past few decades, mounting evidence has shown that lncRNAs play key roles in regulating proliferation, metastasis, the cell cycle and programmed death in cancers (11, 12). For example, we showed that lncRNA LUCAT1 could promote proliferation in colon cancer (13). Recently, many researchers also found that lncRNAs, namely, LINC00618, could play a role in the ferroptosis process in cancer; this lncRNA was found to accelerate ferroptosis in an apoptosis-dependent manner (14). Similarly, LINC00336 inhibits ferroptosis as a competing endogenous RNA in lung cancer (15). Moreover, recent studies have demonstrated that lncRNA GABPB1-AS1 regulates erastin-induced ferroptosis with GABPB1 in HepG2 hepatocellular carcinoma (16). However, current studies screening ferroptosis-related lncRNAs (FRLs) in colon cancer are limited. Accordingly, it is important to identify key FRLs with prognostic significance in colon cancer patients.

In this study, we obtained RNA sequencing (RNA-seq) data from a colon adenocarcinoma (COAD) dataset and ultimately identified four differentially expressed FRLs and developed a prognostic model. Then, the mechanism of action of FRLs in colon cancer was further analysed by gene set enrichment analysis (GSEA), immunoinfiltration analysis and chemotherapy drug sensitivity analysis. Finally, we also tentatively validated the role of two FRLs with high expression in regulating ferroptosis *in vitro*.

Our findings could help to predict the prognosis of colon cancer patients and provide references for clinical chemotherapy and immunotherapy.

## Materials and Methods

### Data Acquisition

The RNA-Seq data of 437 COAD samples, including 39 normal samples and 398 tumour samples, and corresponding clinical characteristics were downloaded from The Cancer Genome Atlas (TCGA) website (https://portal.gdc.cancer.gov/projects/TCGA-COAD). Then, Ensembl IDs were converted to official gene symbols, and log2 processing of the data was performed. LncRNAs and protein-coding genes were screened by the Ensembl human genome browser GRCh38.

### Identification of Ferroptosis-Related LncRNAs

The list of FRGs was downloaded from FerrDb (http://www.zhounan.org/ferrdb/index.html) and contained 121 validated human FRGs. Subsequently, Spearman correlation coefficients were calculated based on FRGs and lncRNA expression profiles to identify FRLs (|R^2^|>0.4 and p < 0.001) (17).

### Differential Expression Analysis

The limma package (18) was used to screen the lncRNA expression matrix between COAD samples and normal colon samples. The criteria for DElncRNAs were |log _2_(fold change) |>1 and a false discovery rate (FDR) <0.05 (19).

### Construction of the Coexpression Network

To demonstrate the correlation of the FRLs and their corresponding mRNAs, the lncRNA-mRNA coexpression network was constructed by Cytoscape software (version 3.7.2, http://www.cytoscape.org/). Then, a Sankey diagram was plotted to show the degree of correlation between FRLs (risk/protect) and their corresponding mRNAs.

### Construction of Ferroptosis-Related Prognostic Signature

The intersecting genes of FRLs and DElncRNAs were filtered by Cox univariate analysis based on the ‘survival’ R package, defining potential prognostic FRLs (p < 0.001). A total of 398 patients were randomly separated into training or validation cohorts at a 1:1 ratio. Then, least absolute shrinkage and selection operator (LASSO)–Cox regression analysis was applied to these prognostic candidates. Finally, by choosing the optimal penalty parameter λ correlated with the minimum 10-fold cross-validation, we established a four-gene optimal prognostic model. The formula for ferroptosis-related prognostic risk scores for each patient was


$$\text{Risk score}=\Sigma_{1}^{n}\;coef_{i}*x_{i}$$

where x_i_ and coef_i_ represent the expression of each lncRNA and its corresponding coefficient, respectively. According to the median value of the risk score, the patients in the training cohort were divided into low-risk and high-risk groups. The Kaplan–Meier curve was generated by using the ‘survminer’ R package with the log-rank test to compare overall survival (OS) between the high/low-risk group. A receiver operating characteristic curve (ROC) (20) was generated to evaluate the predictive accuracy of the signature *via* the ‘timeROC’ R package. To assess the model feasibility, the risk score was calculated in the validation cohort based on the same formula in the training cohort, and then, the same validation method was performed as above.

### Functional Enrichment Analysis

The genes differentially expressed between the high-risk and low-risk groups were identified (|log_2_(fold change)|>1 and FDR<0.05) with the ‘edgeR’ (21) R package and functionally annotated based on the Gene Ontology (GO) and the Kyoto Encyclopedia of Genes and Genomes (KEGG) with the ‘clusterProfiler’ R package (22) (adjusted p value< 0.05).

### Gene Set Enrichment Analysis

To explore the molecular and biological differences in these two groups, GSEA was implemented between high/low ferroptosis risk score groups based on the KEGG and HALLMARK gene sets from the molecular signature database (https://www.gsea-msigdb.org/gsea/msigdb) used as references *via* the ‘clusterProfiler’ R package (p <0.05 and FDR<0.25) (23). Single-sample GSEA (ssGSEA) was performed on several representative gene sets with the ‘GSVA’ R package.

### Assessment of Immune Cell Infiltration and Immune Microenvironment

The ESTIMATE algorithm was used to assess immune infiltration in COAD patients (24). The difference in immune cell infiltration in the two groups of patients was evaluated using the CIBERSORT algorithm (25). CIBERSORT is an analysis tool using expression data to represent the cell composition of complex tissues based on preprocessed gene expression profiles. LM22 of CIBERSORT defines 22 immune cell subsets obtained from the CIBERSORT web portal (http://CIBERSORT.stanford.edu/).

Finally, TIDE (http://tide.dfci.harvard.edu/) algorithms were used to predict immune checkpoint response inhibitors of PD-1 and CTLA4 in the low- and high-risk score groups (26). p < 0.05 was considered significant.

### Drug Sensitivity Prediction

The ‘pRRophetic’ (27) R package was used to predict the IC50 of chemotherapy drugs; this value indicates the effectiveness of a substance in inhibiting specific biological or biochemical processes.

### Tissue Sample Collection and Colon Cancer Cell Line Culture

All tissue samples were collected from the Gastrointestinal Surgery Department of Xiangya 3rd Hospital, which was approved by the Medical Ethics Committee of the hospital. We acquired informed consent from each involved patient before collection. Ten pairs of samples, including tumour tissues (T) and pericarcinous tissues (N), were obtained from colon cancer patients who underwent tumour resection surgery between October 2020 and August 2021. All samples were maintained at -80°C.

Human intestinal epithelial cells (FHCs) and human colon cancer cell lines (HCT116, HT29, SW480, SW620) were purchased from American Type Culture Collection (ATCC) and these cells were cultured in F-12, McCoy’s 5A or Leibovitz’s L-15 medium (Gibco BRL, United States).

With 10% foetal bovine serum (Gibco BRL, United States) at 37°C, 95% humidity, and a 5% CO2 cell incubator.

### RNA Extraction and Quantitative Real-Time Polymerase Chain Reaction (qRT–PCR)

Total cellular and tissue RNA was extracted from tissues or cell lines using Total RNA Extraction Reagent (10606ES60, Yeasen) based on standard protocols. Then, the obtained RNAs were used for cDNA synthesis with a cDNA synthesis kit (11139ES10, Yeasen). Gene expression was quantified by Roche LightCycler 480 using SYBR Green Master Mix (11201ES03, Yeasen), and the expression levels were calculated with the 2^−ΔΔCt^ method. GAPDH acted as the internal reference for normalization. All primers used for qRT–PCR were synthesized by Tsingke Biotech (Tsingke, China). The primer sequences used are listed in **Supplementary Table 1**.

### Cell Counting Kit-8 (CCK-8) Assay

The cells were seeded in 96-well plates at 5×10^3^ cells/well. Then, the cells were treated with different doses of erastin (10 µM) for 24 h. A CCK-8 assay kit (40203ES60, Yeasen) was used to detect cell proliferation at 450 nm. The average inhibition rate of cell activity at each concentration was calculated following the protocol (28).

### Reactive Oxygen Species (ROS) Detection

To detect the ROS level in SW620 cells, 2′,7′-dichlorofluorescin diacetate (DCFH-DA; 5 μM, Sigma–Aldrich, USA) was added to the L15 medium and incubated for 30 min. Fluorescence images were recorded using fluorescence microscopy.

### Determination of Malondialdehyde (MDA) and Fe2+ Levels

MDA levels were detected by an MDA colorimetric assay kit (cell samples, E-BC-K028-M, Elabscience), and Fe2+ levels were measured using a FerroOrange probe (F374, Dojindo). The above assays were performed strictly following the official protocol.

### Statistical Analysis

The Wilcox test was used to compare the proportion of tumour-infiltrating immune cells. Spearman correlation analysis was used to analyse the correlation between FRGs and FRLs. Differences in the proportions of clinical characteristics were analysed by the chi-squared test. Cox univariate regression analysis and multivariate Cox regression analysis were implemented to define the independent prognostic factor for OS. The predictive accuracy of the prognostic model for OS was evaluated by performing time-dependent ROC curve analysis. R software (version 4.10) was applied for all statistical analyses, and the ‘ggplot2’ (29) package was used for graph visualization. Statistical significance was defined as p <0.05, and all p values were two-tailed.

## Results

### Identification of Ferroptosis-Related Differentially Expressed LncRNAs in COAD

**Figure 1:**
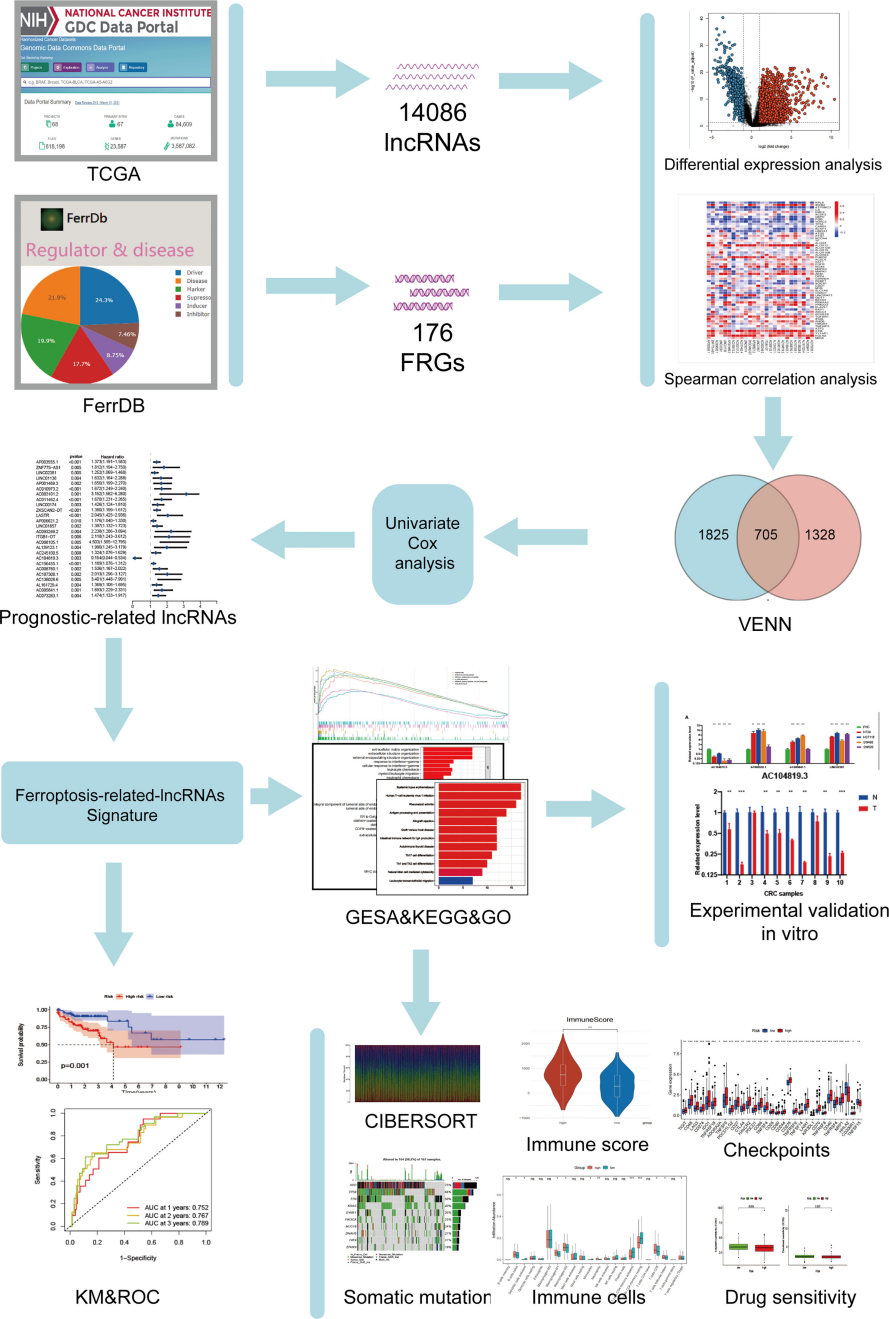
Study flowchart. A total of 14086 lncRNAs and 176 ferroptosis-related genes (FRGs) were obtained from TCGA and FerrDb databases, respectively. Then, 705 ferroptosis-related lncRNAs (FRLs) was identified according to Spearman correlation analysis. Next, univariate COX analysis was applied to screen for the prognostic FRLs. Based on this analysis, a 4-FRL signature was constructed. Subsequently, GSEA, KEGG, GO analyses, immune-related analyses, somatic mutation, and drug sensitivity assays were applied to identify the potential function of this signature. Finally, in vitro validations were conducted to explore the expression and function of these FRLs.

The research flow chart of our study is shown in **Figure 1**. The data for 437 COAD samples were downloaded from the TCGA database (https://portal.gdc.cancer.gov/repository). A total of 14086 lncRNAs and 19604 mRNAs were identified.

**Figure 2:**
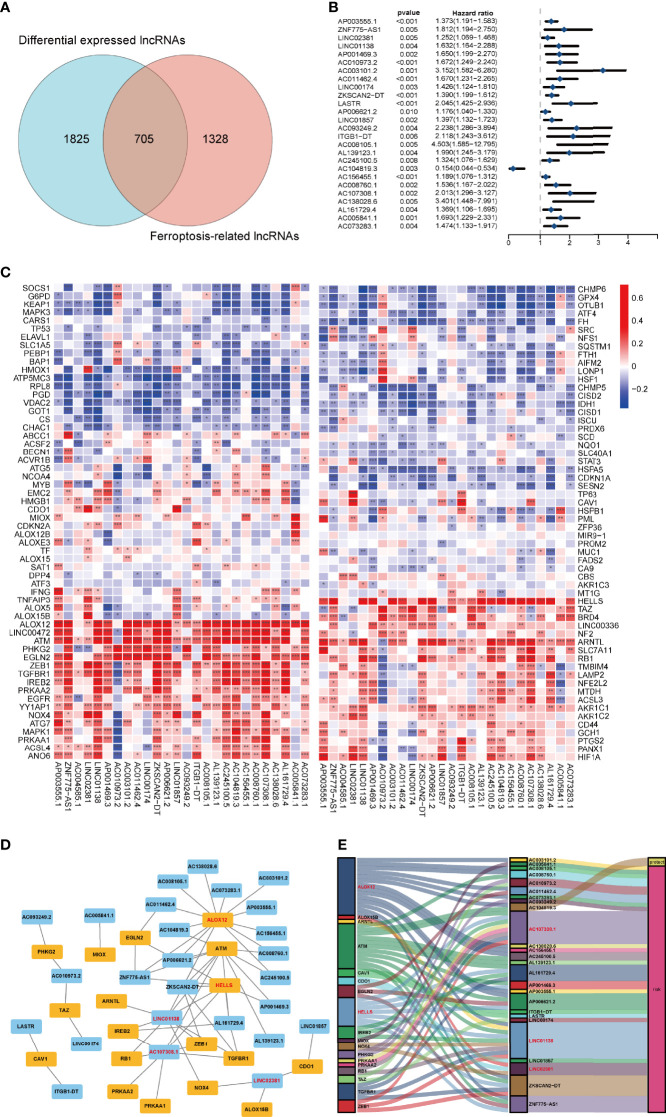
Prognostic analysis of differentially expressed ferroptosis-related lncRNAs and the construction of a coexpression network. **(A)** Venn diagram to identify the common lncRNAs of differentially expressed lncRNAs and ferroptosis-related lncRNAs. **(B)** Forest plots showing the results of the Cox univariate regression analysis approximately 26 prognostic differentially expressed ferroptosis-related lncRNAs. **(C)** The correlation between 26 prognostic ferroptosis-related lncRNAs and 176 ferroptosis-related genes in the TCGA-COAD cohort. The colour of each unit shows the degree of corelation. *p < 0.05, **p < 0.01, and ***p < 0.001. **(D)** Coexpression network of candidate lncRNAs and ferroptosis-related genes. **(E)** The Sankey diagram presents the detail connection between ferroptosis-related lncRNAs and ferroptosis-related genes.

To identify the gene set involved in the process of ferroptosis first, the sequences of FRGs in *Homo sapiens* were downloaded from the FerrDb database (http://www.zhounan.org/ferrdb/) (30); these included 84 ferroptosis driver genes, 89 ferroptosis-related suppressors and 3 ferroptosis-related markers. After the multiannotated genes were screened, a total of 176 FRGs were identified. The details of these genes are documented in **Supplementary Table 2**. Then, Spearman correlation analysis was conducted between lncRNAs in the TCGA database. A PCA map and bar plots showing the distribution of those samples are shown in **Supplementary Figures 1A, B**. FRGs in the FerrDb database were used to determine FRLs. The inclusion parameters were selected as correlation coefficient (|R^2^|) > 0.4 and p value (P)<0.001. In total, 2033 FRLs were defined. Then, we identified 2530 differentially expressed lncRNAs (DELs) in TCGA-COAD samples between normal and tumour tissue (log_2_| FC| > 1, FDR < 0.05), including 1779 upregulated DELs and 751 downregulated DELs. A related volcano map is shown in **Supplementary Figure 1C**. Finally, we identified 705 ferroptosis-related DELs (FRDELs) (**Figure 2A**).

### Identification of Prognostic Ferroptosis-Related Differentially Expressed lncRNAs

To verify the prognostic potential of the FRDELs, these FRDELs were evaluated for prognostic potential by Cox univariate regression analysis using the OS data of COAD patients in the TCGA database. Ultimately, 26 prognostic FRDELs (PFRDELs) in COAD were determined (**Figure 2B** and **Supplementary Figure 1D**). Twenty-five PFRDELs were “risk” genes, while only AC104819.3 could be treated as a “protective” gene. The correlation between 26 PFRDELs and 176 FRGs is shown in **Figure 2C** (the list of these lncRNAs is shown in **Supplementary Table 3**), which implied a reciprocal relationship (the correlation rate and the p value) between each PFRDEL and FRG.

To further evaluate the relationship between these 26 lncRNAs and the representative FRGs, a lncRNA-gene coexpression network was established (**Figure 2D**). Among these FRLs, lncRNA AC107308.1 had a tight linkage with FRGs. A total of 12 genes were coexpressed with lncRNA AC107308.1 (IREB2, NRAS, KRAS, ZEB1, PRKAA2, PRKAA1, TGFBR1, ATM, FBXW7, ANGPTL7, KLHL24, TUBE1). In addition, lncRNA LINC01138 was coexpressed with 10 genes (IREB2, NOX4, ALOX12, ZEB1, TGFBR1, ATM, FBXW7, ANGPTL7, ZNF419, KLHL24), and lncRNA LINC02381 also had a connection with 3 FRGs (ALOX15B, NOX4, CDO1). Among those FRGs, Acid 12-lipoxygenase (ALOX12), a well-known ferroptosis driver (31), had positive coexpression with 14 prognostic FRLs. HELLS is also connected with 7 FRLs, and the details of the coexpression network are shown in **Supplementary Table 4**. Subsequently, we further visualized the prognostic function and discovered the internal connection between PFRDELs and FRGs. We also established a Sankey diagram (32) that showed the relationship among FRLs, FRGs and their roles in COAD (**Figure 2E**).

### Construction and Validation of a FRL Prognostic Model

To check the prognostic value of these FRDELs, the samples from TCGA-COAD database were classified randomly into two groups: a training group and a validation group. The clinical characteristics of the samples in the two groups are shown in **Table 1**.

**Table 1:** The clinical characteristics of colon cancer patients in the training and validation group.

Characteristics	Training group	%	Validation group	%	P-value
	No.		No.		
**Age**	—		—		—
<=60	49		45		>0.05
>60	112		125		—
**Gender**	—		—		—
Male	83		94		>0.05
Female	78		76		—
**AJCC Stage**	—		—		—
I	30		28		>0.05
II	61		73		—
III	44		42		—
IV	26		27		—
**T stage**	—		—		—
T1	3		4		>0.05
T2	28		30		—
T3	115		115		—
T4	15		21		—
**N stage**	—		—		—
N0	94		105		>0.05
N1	37		38		—
N2	30		27		—
**M stage**	—		—		—
M0	135		143		>0.05
M1	26		27		—

**Figure 3:**
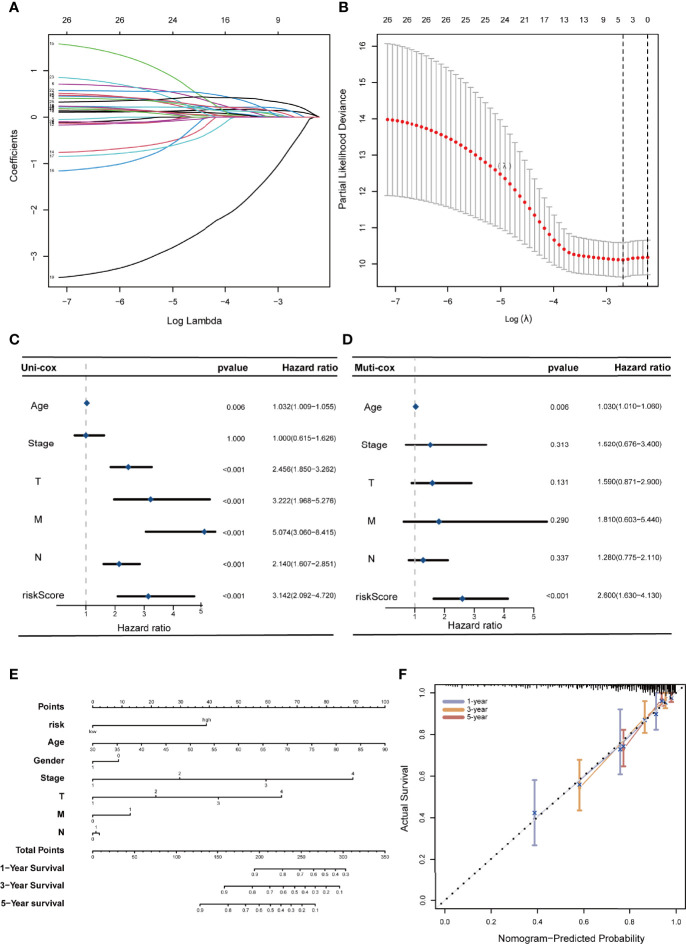
Construction of a 4-ferroptosis-related-lncRNA signature and the analysis of independent prognostic potential. **(A, B)** cvfit and lambda curves showing the least absolute shrinkage and selection operator (LASSO) regression was performed with the minimum criteria. **(C, D)** Results of the univariate Cox regression analysis and multivariate Cox regression analysis regarding OS of the 4-ferroptosis-related-lncRNAs signature. **(E)** The nomogram to predict the 1-year, 3-year, and 5-year overall survival rate of colon cancer patients. **(F)** The calibration curve for evaluating the accuracy of the nomogram model. The dashed diagonal line in grey colour represents the ideal nomogram.

A prognostic risk evaluation model based on only 4 FRLs was then constructed using the optimal penalty parameter (λ) for the LASSO model from the abovementioned 26 PFRDEL lesions in the training group. The cvfit and lambda curve are shown in **Figures 3A, B**. In this model, each COAD patient in the TCGA database was assigned a risk score using the following formula: Risk Score=AC104819.3*(-0.52383)+AP003555.1*0.12181+AC005841.1*0.25406+LINC02381*0.10087 (Note: the name of lncRNA indicates their expression level in TCGA database). Cox univariate and multivariate regression analyses were performed to evaluate the independent predictive potential of this signature. First, Cox univariate regression analysis demonstrated that the risk score of this signature was associated with the OS rates of COAD patients (p=0.011; **Figure 3C**). Furthermore, multivariate Cox regression analysis revealed that only this 4-FRL risk signature and age could act as an independent prognostic factor for predicting the OS rates of COAD patients in the TCGA database (p < 0.001; **Figure 3D**). The predictive nomogram calculated the likelihood of survival of those patients by adding up the scores identified on the points scale for the many related factors. The 1-, 3- and 5-year OS rates could be predicted accurately when compared with those of the ideal predictive model (**Figures 3E, F**).

**Figure 4:**
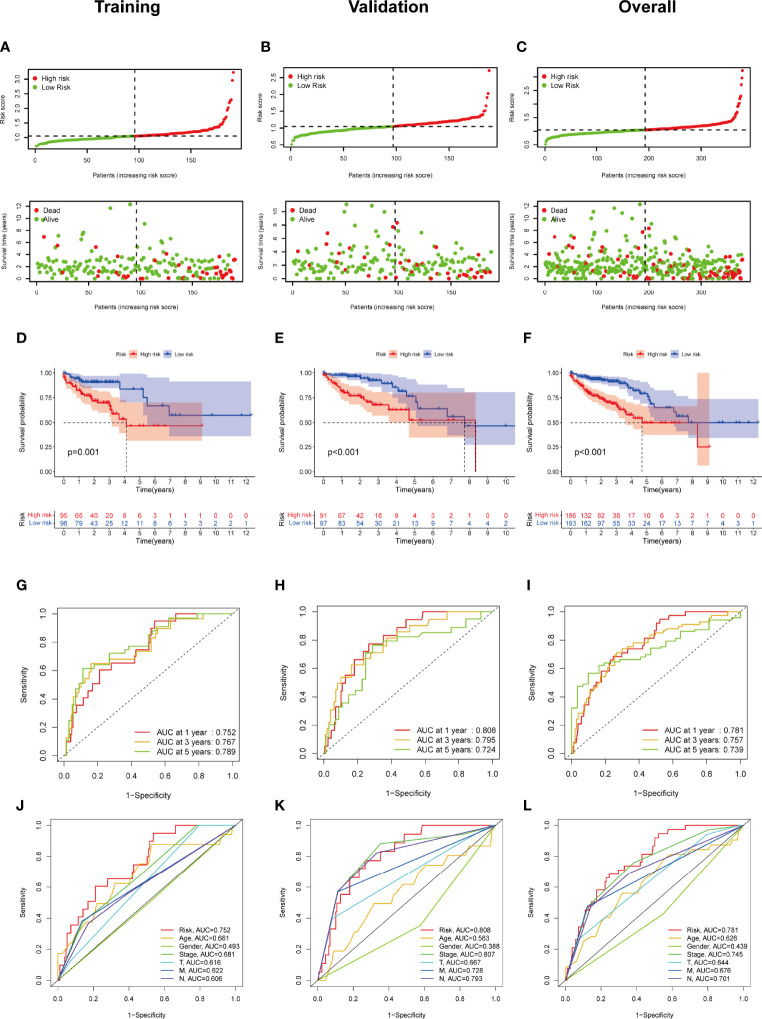
Construction and validation of the ferroptosis-related lncRNA signature model in the training cohort, validation and overall groups. **(A–C)** The distribution of the risk scores and the distributions of overall survival status and risk score in the training, validation and overall groups. **(D–F)** The Kaplan–Meier curves for survival status and survival time in the training, validation and overall groups. **(G–I)** The receiver operating characteristic (ROC) curve shows the potential of the prognostic ferroptosis-related lncRNAs signature in predicting 1-, 2-, and 3-year overall survival (OS) in the training, validation and overall groups. **(J–L)** AUC of ROC curves comparing the prognostic accuracy of the risk score and other prognostic factors in the training, validation and overall groups.

Subsequently, we evaluated the prognostic value of this 4-FRL model. Then, the samples in the training group were classified into high-risk and low-risk groups according to the median value of the risk scores. The distribution of the risk scores and the distribution of the OS status were visualized to show that those samples of the above two risk groups were reasonably distributed (**Figure 4A**). Kaplan–Meier survival analysis was then used to show that the OS rate of COAD patients in the high-risk group was worse than that in the low-risk group (**Figure 4D**). A time-dependent ROC curve was also generated in the training group. The areas under the curve (AUCs) were maintained at more than 0.75 at the 1-year, 3-year and 5-year points (**Figure 4G**). An ROC curve was also constructed to validate the outstanding prognostic accuracy of this signature compared to other clinicopathological characteristics (**Figure 4J**). To further evaluate the predictive efficacy of this 4-lncRNA signature, the distribution figures, heatmaps, Kaplan–Meier survival analysis and time-dependent ROC analysis were double validated in both the validation group and the overall group. The samples of the above two risk groups were also reasonably distributed in the validation group (**Figures 4B, E, H, K**) and the overall group (**Figures 4C, F, I, L**). It is obvious that individuals from the high-risk group may have higher mortality rates than low-risk individuals.

### Relationship Between the 4-FRL Signature and the Clinicopathological Characteristics in COAD Patients

**Figure 5:**
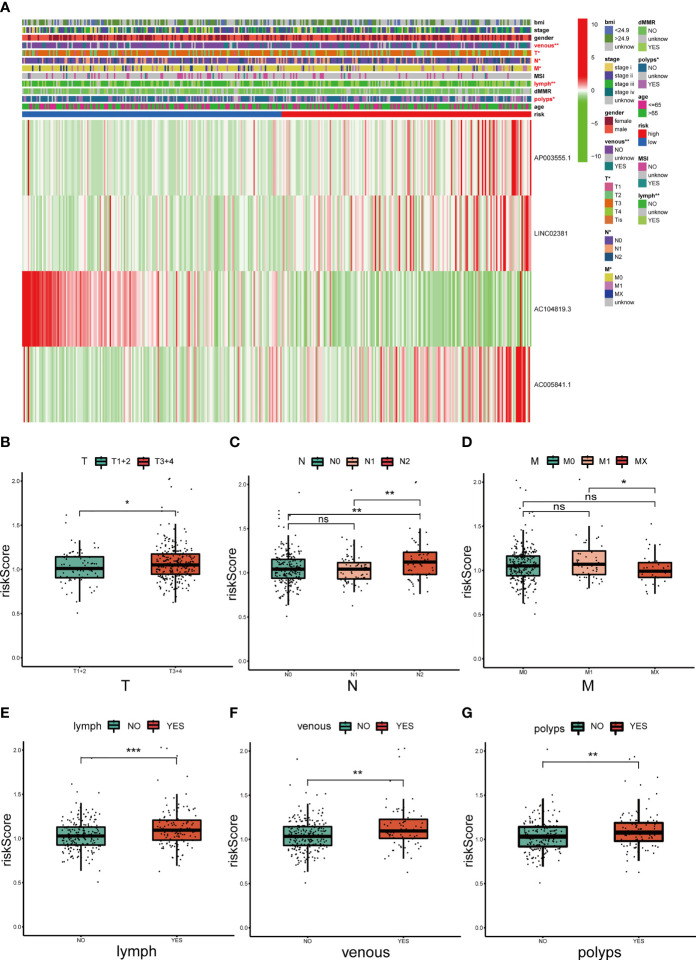
Correlation analysis between the prognostic signature and different clinicopathological characteristics in the TCGA cohort. **(A)** The heatmap depicting the distribution of 12 different clinicopathological characteristics with the risk scores of each patient based on the signature. **(B–G)** The histogram depicting the significant difference of the risk scores in colon cancer patients stratified by T stage, N stage, M stage, lymph invasion, venous invasion and polyp history. *p < 0.05, **p < 0.01, and ***p < 0.001. ns, No significance.

Three lncRNAs in our signature were considered risk lncRNAs, and they were upregulated in the high-risk group in the TCGA-COAD database. Only AC104819.3 was a protective lncRNA that was downregulated in the high-risk group (**Figure 5A**). We compared the differences in clinicopathological characteristics between the two risk subgroups. Interestingly, there were significant differences in tumour stage (p<0.01), T stage (p<0.01), N stage (p<0.001), M stage (p<0.01), microsatellite stability (p<0.05), venous invasion (p<0.001) and lymph invasion (p<0.01) (**Figure 5A**) between these two groups, and the above clinical characteristics were also compared separately in **Figures 5B–G**. The high-risk group exhibited advanced T and N stages compared with those of the low-risk group, and lymph and venous invasion were more frequent in the high-risk group. Interestingly, we also noticed that more patients from the high-risk group had a history of polyps. In sum, these results indicated that this 4-lncRNA signature has outstanding potential for predicting prognosis in COAD patients by evaluating their risk score by related gene expression level.

### Discovery of Molecular Functions and Pathways by GSEA, GO and KEGG Analysis

**Figure 6:**
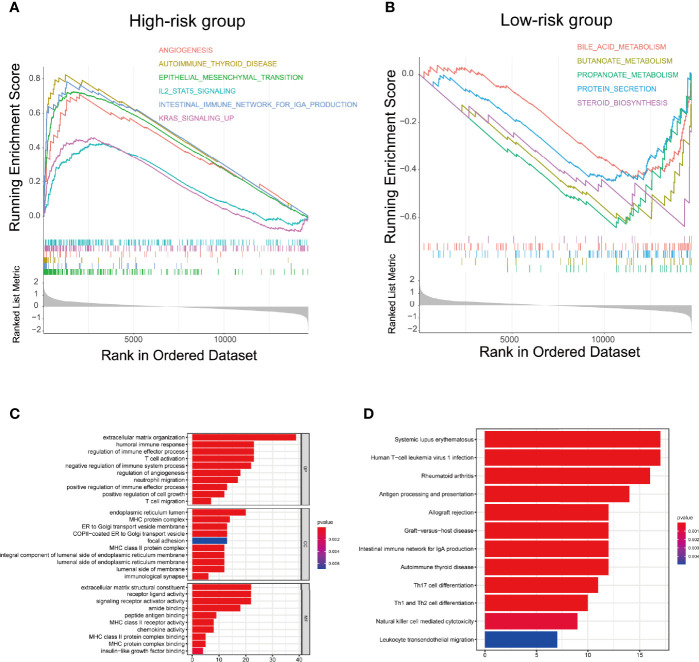
Biological functional and pathway enrichment analysis of high-risk group and low-risk group based on the ferroptosis-related lncRNA prognostic signature. **(A)** GSEA showing significant enrichment of immune-related pathways and cancer proliferation pathways in the high-risk colon cancer patients. **(B)** GSEA showing significant enrichment of metabolism related pathways in the low-risk colon cancer patients. **(C)** GO analysis showing many immune-related biological processes were enriched. **(D)** KEGG analysis showing many immune-related pathways and cancer proliferation pathways were enriched.

To explore the underlying difference in biological functions and signalling pathways between the different risk groups classified by the 4-FRL signature. GSEA was performed. The results showed that many cancer proliferation pathways were enriched in the high-risk group, such as angiogenesis-related pathways and the KRAS pathway. Many immune-related pathways were also involved, such as the autoimmune thyroid disease, the IL2 pathway, and the intestinal immune network (**Figure 6A**). Moreover, many metabolic pathways were enriched in the low-risk group, such as bile acid metabolism, butanoate metabolism, propanoate metabolism and drug metabolism (**Figure 6B**). Interestingly, some pathways related to drug resistance, such as KESHELAVA multiple drug resistance, cisplatin resistance and the MAPK pathway, were also enriched. The details of the GSEA results are listed in **Supplementary Table 5**. We further investigated the differences in biological processes and pathways in differentially expressed genes(DEGs) between the two risk groups. DEGs between the high-risk group and the low-risk group were determined by the cut-off of log_2_| FC| > 1 and FDR < 0.05, and annotation GO enrichment analysis and KEGG pathway analysis were then performed (p<0.05). The KEGG analysis showed that many immune-related pathways were significantly enriched, including systemic lupus erythematosus, Th1, Th2 and Th17 cell differentiation, antigen processing and presentation, which were similar to the results of GSEA (**Figure 6C**). GO analysis was conducted and indicated the enrichment of biological process (BP), molecular function (MF), and cell component (CC). The results of these three analyses are presented in **Figure 6D**. In summary, these results suggested that the risk score of the 4-lncRNA signature was mainly related to tumour metastasis, tumour immunity, biological metabolism and drug resistance in colon cancer.

### Immune-Related Analysis of COAD Patients Using the Prognostic Signature

**Figure 7:**
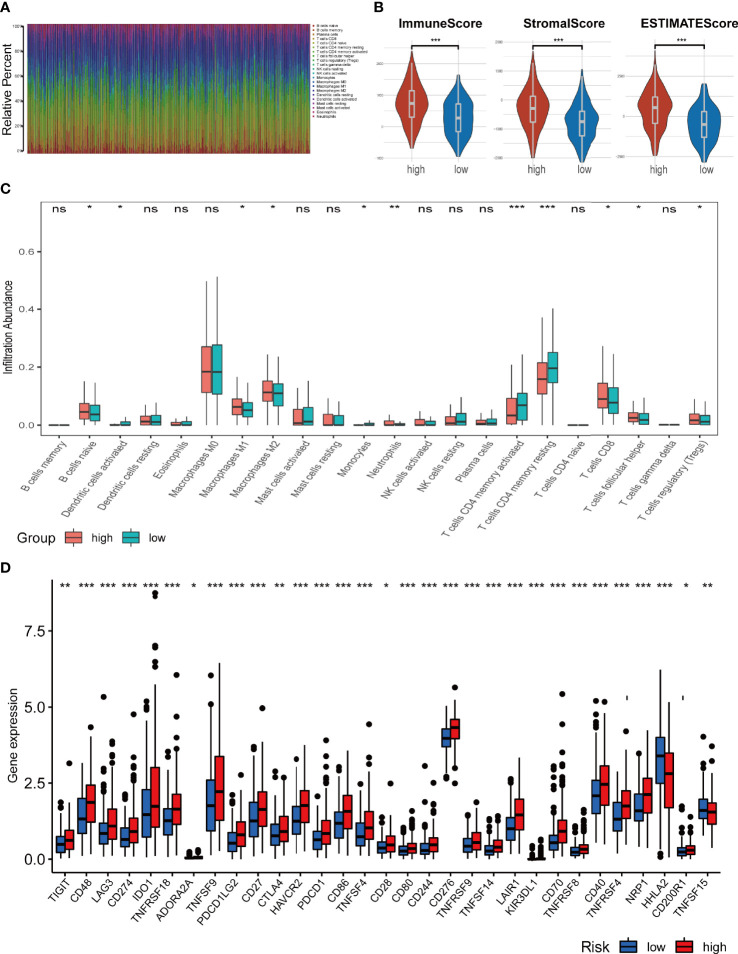
Analysis of immune cell infiltration landscape in colon cancer patients. **(A)** Bar graphs exhibiting the distribution of tumour-infiltrating immune cells between the high-risk and low-risk groups based on the ferroptosis-related-lncRNAs signature. **(B)** Stroma, immune, and ESTIMATE scores in the high-risk and low-risk groups in colon cancer patients. **(C)** The boxplots for the comparison of the 22 immune cells between the high-risk and low-risk groups in the colon cancer patients. **(D)** The boxplots for the comparison of the immune checkpoints genes between the high-risk and low-risk groups in the colon cancer patients. *p < 0.05, **p < 0.01, and ***p < 0.001, ns, No significance.

To further explore the relationship between the ferroptosis-related signature and antitumour immunity in COAD patients, we identified the immune cell infiltration landscape of all patients with COAD from the TCGA database using the CIBERSORT algorithm. The proportion of each typical immune cell is shown in **Figure 7A**. To identify the differences in infiltrating immune cells between the high-risk and low-risk groups, the stromal score (substrate cells in the tumour tissue), immune score (immune cell infiltration in the tumour tissue) and estimate score (the summation of stromal and immune scores from individual cases) were compared, and these scores were all significantly higher in the high-risk group (p<0.001) (**Figure 7B**). Moreover, we also compared the proportion of each immune cell between the high-risk and low-risk groups and found that naive B cells, activated dendritic cells, M1 and M2 macrophages, neutrophils, monocytes, resting CD4 memory T cells, activated CD4 memory T cells, CD8 T cells, follicular helper T cells and regulatory T cells were significantly different between the two groups (**Figure 7C**).

We also compared the expression levels of immune checkpoint genes in the high-risk and low-risk groups. As shown in **Figure 7D**, 30 checkpoint genes were significantly different between the two groups. Among these, 28 genes presented with high expression in the high-risk group, including many validated effective immunotherapy targets, such as PDCD1 (PD-1), CD274 (PD-L1) and CTLA4. HHLA2 and TNFSF15 expression was lower in the low-risk group than that in the high-risk group. Altogether, the relationships between the risk scores calculated by the 4-lncRNA signature and immune infiltration cells were evaluated, and the results indicated that the risk level of those COAD patients was associated with those immune infiltration cells.

### Cancer-Related Gene Mutation and Drug Sensitivity in the 4-Ferroptosis-Related LncRNA Signature

**Figure 8:**
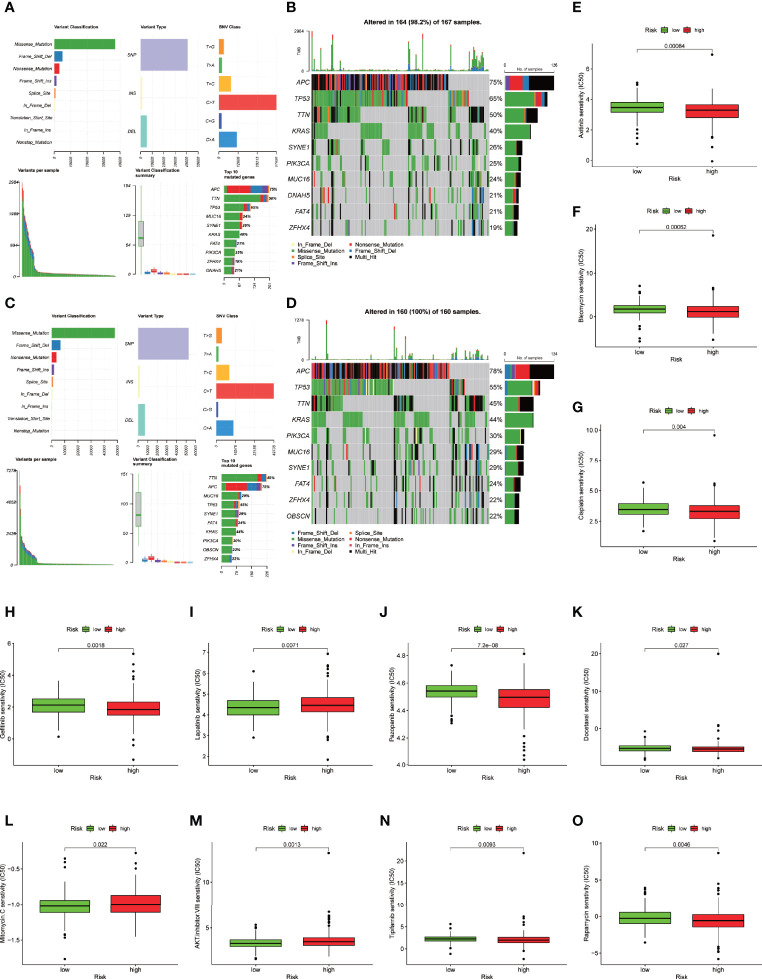
Somatic mutation analysis and drug sensitive prediction in colon cancer patients. **(A–D)** MAF-summary plots and oncoplots of the somatic mutation showing the difference between the high-risk group and low-risk group in colon cancer patients. **(E–O)** Boxplot showing the mean differences in estimated IC50 values of 11 representative drugs (cisplatin, docetaxel, bleomycin, axitinib, gefitinib, pazopanib, rapamycin, tipifarnib, lapatinib, mitomycin C, AKT inhibitor VIII) between the two risk groups.

To identify the difference in cancer-related gene mutations between the high-risk and low-risk groups, we first counted the gene mutation in each group. General information on representative gene mutations in both groups is shown in **Figures 8A–D**. Genes such as APC (75%), TP53 (65%), TNN (50%), KRAS (40%) and SYNE1 (26%) had the top five mutation frequencies in the high-risk group. APC (78%), TP53 (55%), TNN (45%), KRAS (44%) and PIK3CA (30%) were the top five genes with the highest mutation frequencies in the low-risk group. Generally, anti-oncogenes, such as TP53, had a relatively higher mutation rate in the high-risk group (65% vs. 55%), while oncogenes such as MUC16 presented a relatively lower mutation rate in the high-risk group (24% vs. 29%).

To further explore the difference in the two risk groups about the drug resistance potential. We compared the estimated IC50 levels of 138 chemotherapy drugs or inhibitors in the two groups. Among those, 11 representative drugs are shown in **Figures 8E–O**. We found that cisplatin, docetaxel, bleomycin, axitinib, gefitinib, pazopanib, rapamycin and tipifarnib may be candidate drugs for treating patients in the high-risk group. Lapatinib, mitomycin C, and AKT inhibitor VIII may not be ideal for patients in the high-risk group.

### Validation of FRL Expression

To evaluate the protein-coding ability of these FRLs, we used PhyloCSF (33) to determine whether these FRLs are likely to represent conserved protein-coding regions. As shown in **Supplementary Figure 2**. AP003555.1, AC104819.3 and LINC02381 with negative scores were retained as potential noncoding RNAs (34), while AC005841.1 may have the potential to encode 4 kD short peptides because it obtained one exon with a positive score.

**Figure 9:**
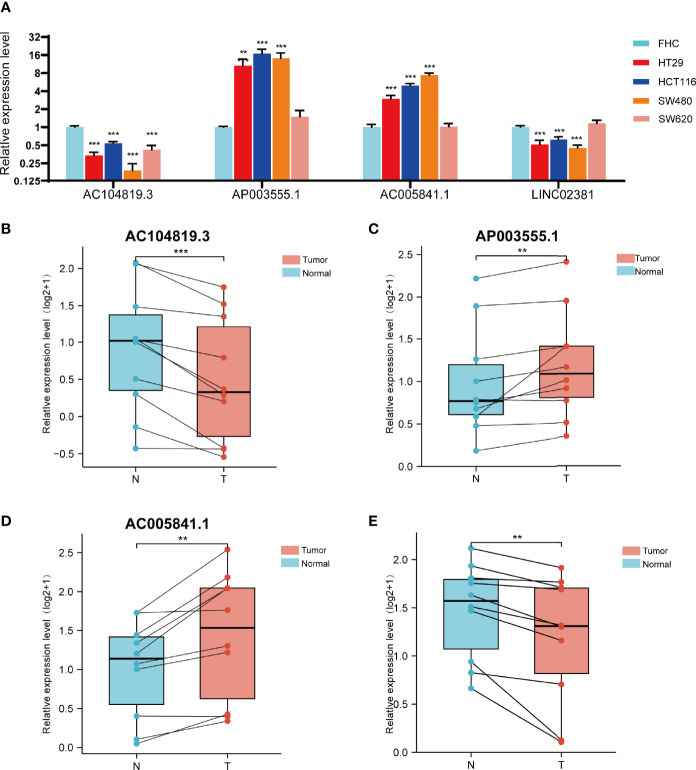
Validation of the expression level of the four ferroptosis-related lncRNAs in cell lines and tissues. **(A)** Expression analysis of four ferroptosis-related lncRNAs in four colon cancer cell lines (HT29,HCT116,SW480,SW620) with FHC lines (established from normal foetal colonic mucosa). **(B–D)** Expression analysis of AP003555.1, AC104819.3, AC005841.1 and LINC02381 in 10 pairs of colon cancer tissue samples. **p < 0.01, and ***p < 0.001.

We further evaluated the expression levels of these 4 prognostic FRLs. We tested their expression level in the cell lines. As shown in **Figure 9A**, compared with those in the FHC line (established from normal fetal colonic mucosa), AP003555.1 and AC005841.1 were expressed at relatively higher levels in colon cancer cell lines (including HT29, HCT116, SW480, and SW620), but AC104819.3 and LINC02381 exhibited the opposite trend. We also validated the expression levels of these 4 lncRNAs in sample pairs retreated from colon cancer patients in our hospital. Similar expression trends were observed in clinical samples (**Figures 9B–E**). AP003555.1 and AC005841.1 showed higher expression levels in tumour tissues (T) than in pericarcinous tissues (N). These results further verified the correctness of the above bioinformatics research (**Supplementary Figure 1D**).

### AP003555.1 and AC005841.1 Regulated Erastin-Induced Ferroptosis

**Figure 10:**
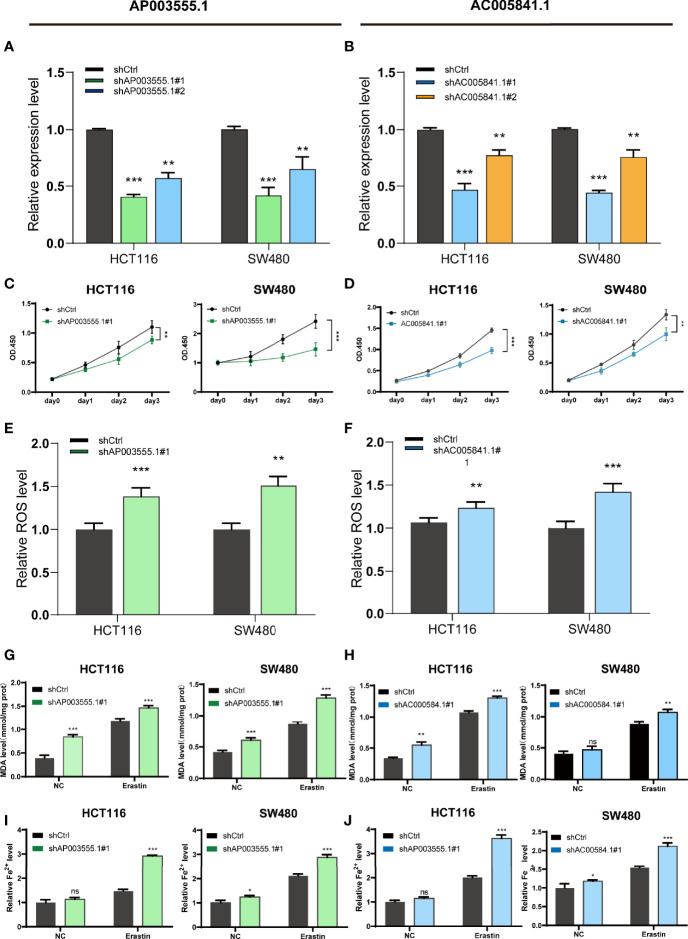
Ferroptosis regulation of AP003555.1 and AC005841.1. **(A, B)** Relative expression level of AP003555.1 and AC005841.1 after transfection with the corresponding shRNA. **(C)** The cell proliferation ability of HT116 and SW480 cells after the knockdown of AP003555.1. **(D)** The cell proliferation ability of HT116 and SW480 cells after the knockdown of AC005841.1. **(E, F)** The comparison of erastin-induced ROS in the treatment and control groups. **(G–J)** The ferroptosis process was evaluated by detecting MDA and Fe2+ levels in the non-erastin-induced and erastin-induced groups. *p < 0.05, **p < 0.01, and ***p < 0.001, ns, No significance.

As mentioned previously, compared with FHC, the expression of both AP003555.1 and AC005841.1 was significantly upregulated in the CRC cell lines, especially in HCT116 and SW480 cells. AC104819.3 and LINC02831 were slightly downregulated in CRC cells. Thus, AP003555.1 and AC005841.1 were chosen for further analysis. To further elucidate the potential function of these two FRLs, we implemented the stable knockdown of AP003555.1 and AC005841.1 using short hairpin RNAs in HCT116 and SW480 cells. The transfection efficiency was confirmed by qRT–PCR (**Figures 10A, B**). Then, a Cell Counting Kit-8 (CCK-8) assay was used to evaluate their roles in regulating the proliferation of colon cancer cells. The knockdown of AP003555.1 and AC005841.1 significantly inhibited cell proliferation in HCT116 and SW480 cells compared to their control groups (**Figures 10C, D**). Ferroptosis is mainly characterized by the accumulation of ROS. ROS levels were clearly observed after HCT116 and SW480 cells were treated with 10 µM erastin (ferroptosis activator). As expected, erastin-induced ROS production was increased after the knockdown of both AP003555.1 and AC005841.1 (**Figures 10E, F**). Then, malondialdehyde (MDA) and Fe^2+^ levels were measured by MDA and FerroOrange assay kits, and MDA and Fe^2+^ levels were remarkably increased after AP003555.1 and AC005841.1 silencing after treatment with 10 µM erastin in CRC cells (**Figures 10G–J**).

## Discussion

Currently, many studies have focused on the roles of lncRNAs in the ferroptosis of cancer (35). The identification of FRLs is indispensable in searching for potential cancer targets. However, studies on FRLs in colon cancer remain limited.

In this study, we comprehensively analysed the expression profiles of 176 validated FRGs in humans provided by the latest online FerrDb database and screened out differentially expressed FRLs. Subsequently, the prognosis of each patient in the TCGA database and the expression profile of these FRLs were analysed. The results identified 26 prognostic FRLs. Then, a lncRNA-gene coexpression network was established, and we noticed that ALOX12 has high correlations with 12 prognostic FRLs. ALOX12 has been shown to play an important role in inflammation and oxidation (36). This enzyme can elevate the levels of mitogenic metabolites in cancer cells and thus increase the proliferation rate of cancers (37, 38). Recently, Bo Chu et al. found that ALOX12 inactivation diminishes p53-mediated ferroptosis induced by ROS stress and abrogates p53-dependent inhibition of tumour growth (17), which means that ALOX12 might function as a hub gene with a deep connection with many essential FRLs. We also noticed that LINC02831 had high correlations with 3 mRNAs (NOX4, ALOX15B, and CDO1). LINC02381 is an oncogene that has been validated by many cancer researchers. For example, LINC02381 can promote cell proliferation and migration by targeting miR-133b in cervical cancer (39), and it inhibits gastric cancer by regulating the wnt pathway (40). However, how LINC02381 is involved in regulating ferroptosis still needs further exploration. Significantly, we noticed that LINC02381 expression was lower in tumour samples than in nontumour samples; however, it still functions as a “risk” lncRNA in colon cancer.

Furthermore, a novel prognostic 4-lncRNA model was created. Specifically, this signature is relatively easier to use in the clinic than many other identified signatures because it only included 4 lncRNAs, and it also exhibited a greater ability to predict the prognosis of colon cancer patients than the traditional TNM stage. Many adverse events, such as venous invasion or lymphatic metastasis, could also be foreseen by evaluating the risk score of patients using this model. We divided colon cancer patients into a high-risk group and a low-risk group based on their risk scores calculated by the formula of this prognostic model. To further evaluate the mechanism of how this signature regulates the process of colon cancer. GSEA was then conducted. The results revealed that the pathway of angiogenesis ranks high in the high-risk group, and angiogenesis (the formation of new blood vessels) has been proven integral to cancer development (41). Cancer metastasis pathways such as cell adhesion or epithelial-mesenchymal transition (EMT) (42) were also enriched. The relationship between ferroptosis and the immunosuppressive microenvironment is a contentious issue (43). We noticed that many immune-related hallmarks were enriched, such as the intestinal immune network or IL2-STAT5 pathway, and we can reasonably assume that tumour immunity is closely related to ferroptosis in colon cancer. Lipid peroxidation has been considered a vital process in ferroptosis (44). Therefore, researchers also believe that aberrant metabolic and biochemical processes contribute to ferroptosis (45). Many metabolic pathways, including fatty acid metabolism, were also enriched. KEGG enrichment analysis and GO enrichment analysis, including BP, MF and CC, were also performed, and the enrichment pathway results were relatively similar to the GSEA results. In summary, we may infer from the results above that ferroptosis was inhibited in the high-risk group through some immune-related pathways. Therefore, colon cancer could initially develop in these patients.

Previous studies have also suggested that ferroptosis is closely related to tumour immunity. It is also considered immunogenic cell death (46). Wang et al. verified that CD8+ T cells could induce ferroptosis in tumour cells (47). Some studies also found that prostaglandin E2 (PGE2) facilitates tumour immune evasion (48, 49). However, no study has reported a direct relationship between ferroptosis and immune cell infiltration in colon cancer. After many immune-related pathways were enriched in our GSEA, we calculated the proportion of different types of tumour-infiltrating immune cells in colon cancer from TCGA database using CIBERSORT. As expected, we found that the high-risk group showed significantly higher immune, stromal and ESTIMATE scores than the low-risk group. Previous studies revealed that high immune and stromal scores as well as high infiltration of macrophages were associated with poor prognosis, which was in accordance with our results (50). Furthermore, patients in the high-risk group also presented relatively low expression levels of immune cells such as monocytes or dendrites, and immature immune cells such as naive B cells or immunosuppressive cells such as regulatory T cells were expressed at higher levels in the low-risk group. CD4 T cell responses are essential in the cancer immune cycle, and both significantly influence the clinical outcome (51). We witnessed a notable decrease in CD4 T cells in the high-risk group, and we assume that the CD4 function of colon cancer patients might be relatively inhibited or slowed in the high-risk group. The expression levels of many immune checkpoints, such as PD-1, PD-L1, and CTL4A, were higher in the high-risk group than in the low-risk group. Therefore, these patients might benefit from many immune checkpoint blockades (52), which might improve the prognosis of high-risk patients by enhancing their immunoreactivity or inducing ferroptosis.

Additionally, we evaluated the expression level of these 4 PFRDELs in our signature. The expression trend was basically consistent with the prediction of the previous bioinformatic analysis. Finally, many ferroptosis-related assays were conducted to elucidate the potential mechanisms of two lncRNAs in our signature, AP003555.1 and AC005841.1, which were proven to regulate ferroptosis in a ferroptosis-dependent manner. However, the role of LINC02381 in ferroptosis needs further exploration because no significant changes in MDA and Fe^2+^ levels were observed. Considering that the role of LINC02381 in cancer remains disputed (40, 41), we suggest that LINC02381 may work together with other genes in regulating ferroptosis. There are still some limitations that must be addressed. First, external validation was missing due to the lack of expression profiles of lncRNAs and OS data in other databases. Therefore, validation could only be performed *via* the TCGA database. Second, even though the expression levels of all 4 lncRNAs were checked by qRT–PCR in 10 pairs of clinical samples and 5 colon cancer cell lines, there were still not sufficient samples available, and more samples would be helpful to make the evidence more solid. Finally, the underlying mechanism of how these lncRNAs affect ferroptosis remains unknown. Further research on the relationship between these lncRNAs and FRGs is necessary.

## Conclusion

Our study constructed a robust prognostic predictive model with only 4 FRLs, which, compared to other traditional clinicopathologic signatures, is relatively easy to test in patients. The relationship between our risk model and the immune landscape was preliminarily ascertained. The findings of our study offer many useful insights in predicting the prognosis of colon cancer patients and may even assist their treatment in clinical practice.

## Data Availability Statement

The original contributions presented in the study are included in the article/**Supplementary Material**. Further inquiries can be directed to the corresponding authors.

## Ethics Statement

The studies involving human participants were reviewed and approved by Medical Ethics Committee of the Thrid Xiangya hospital, Central South University. The patients/participants provided their written informed consent to participate in this study.

## Author Contributions

ZW, ZL, and CL contributed to conception and design of the study. ZW and ZL organized the database. ZL performed the statistical analysis. ZW wrote the first draft of the manuscript. ZW and ZL wrote sections of the manuscript. All authors contributed to manuscript revision, read, and approved the submitted version

## Funding

This work was supported by the Wisdom Accumulation and Talent Cultivation Project of the Third Xiangya Hospital of Central South University (No. YX202107).

## Conflict of Interest

The authors declare that the research was conducted in the absence of any commercial or financial relationships that could be construed as a potential conflict of interest.

## Publisher’s Note

All claims expressed in this article are solely those of the authors and do not necessarily represent those of their affiliated organizations, or those of the publisher, the editors and the reviewers. Any product that may be evaluated in this article, or claim that may be made by its manufacturer, is not guaranteed or endorsed by the publisher.

## Supplementary Material

Supplementary Figure 1 Identification of differentially expressed ferroptosis-related lncRNAs in COAD. **(A, B)** The PCA map and the bar plots showing the distribution of colon cancer samples. **(B)** The volcano map of lncRNAs differentially expressed in COAD. **(D)** The heatmap of these 26 lncRNAs showing the expression level of each lncRNA in each patient.

Supplementary Figure 2 The results of 4 FRLs in PhyloCSF.

Supplementary Table 1 The list of primer sequences used in our study.

Supplementary Table 2 The details of all of the ferroptosis-related genes.

Supplementary Table 3 The table of differentially expressed lncRNAs and ferroptosis-related lncRNAs.

Supplementary Table 4 The list of genes involoved in the coexpression network.

Supplementary Table 5 The list of pathways enriched in GSEA.
