# Supplementary figures and images for: Identification and Validation of Ferroptosis-Related LncRNA Signatures as a Novel Prognostic Model for Colon Cancer
Source: Front Immunol. 2022 Jan 26;12:783362. doi: 10.3389/fimmu.2021.783362 (PMC8826443; doi:10.3389/fimmu.2021.783362)

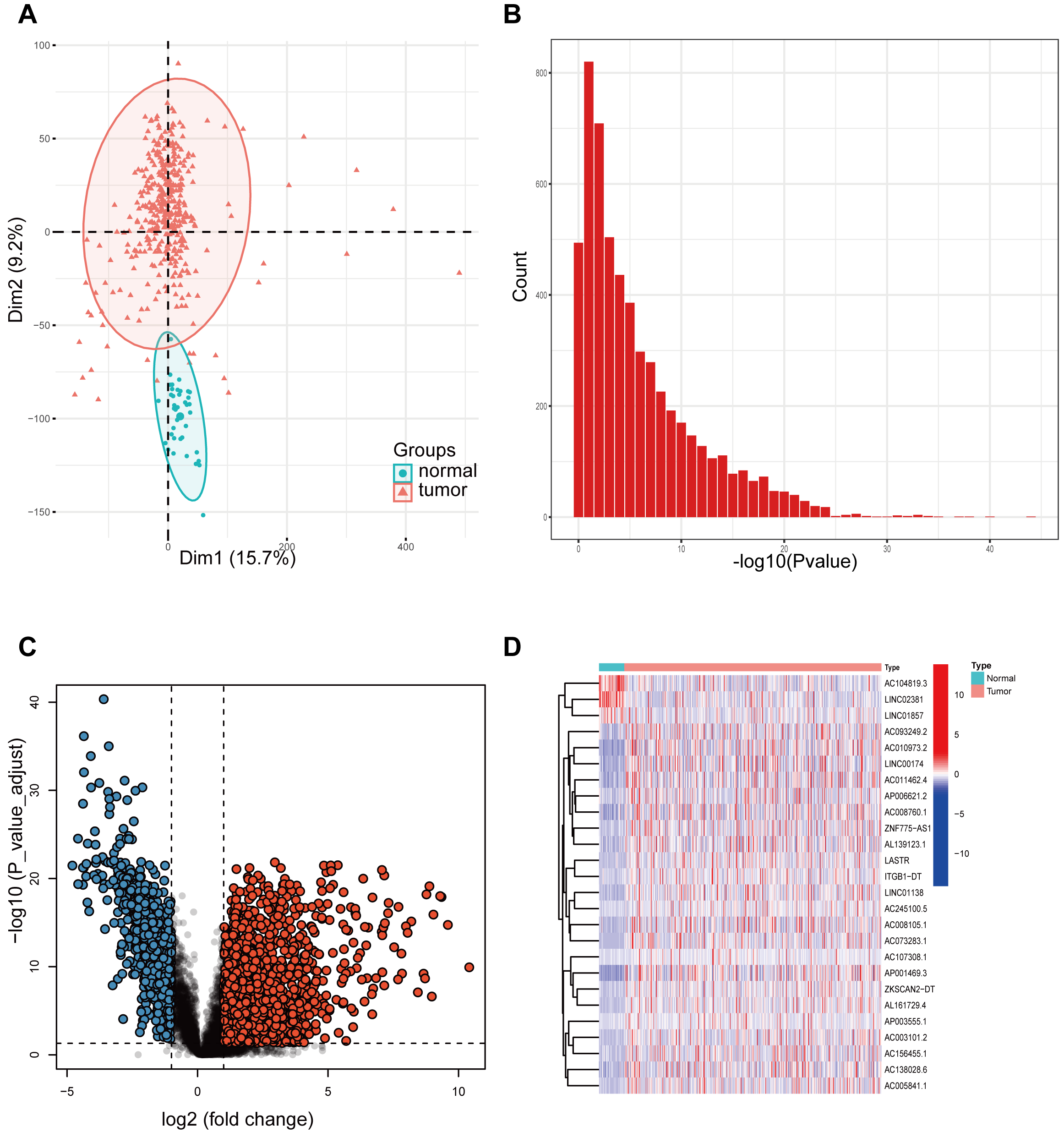

Supplement: Supplementary Figure 1 — Identification of differentially expressed ferroptosis-related lncRNAs in COAD. (A, B) The PCA map and the bar plots showing the distribution of colon cancer samples. (B) The volcano map of lncRNAs differentially expressed in COAD. (D) The heatmap of these 26 lncRNAs showing the expression level of each lncRNA in each patient. [file Image_1.tif]

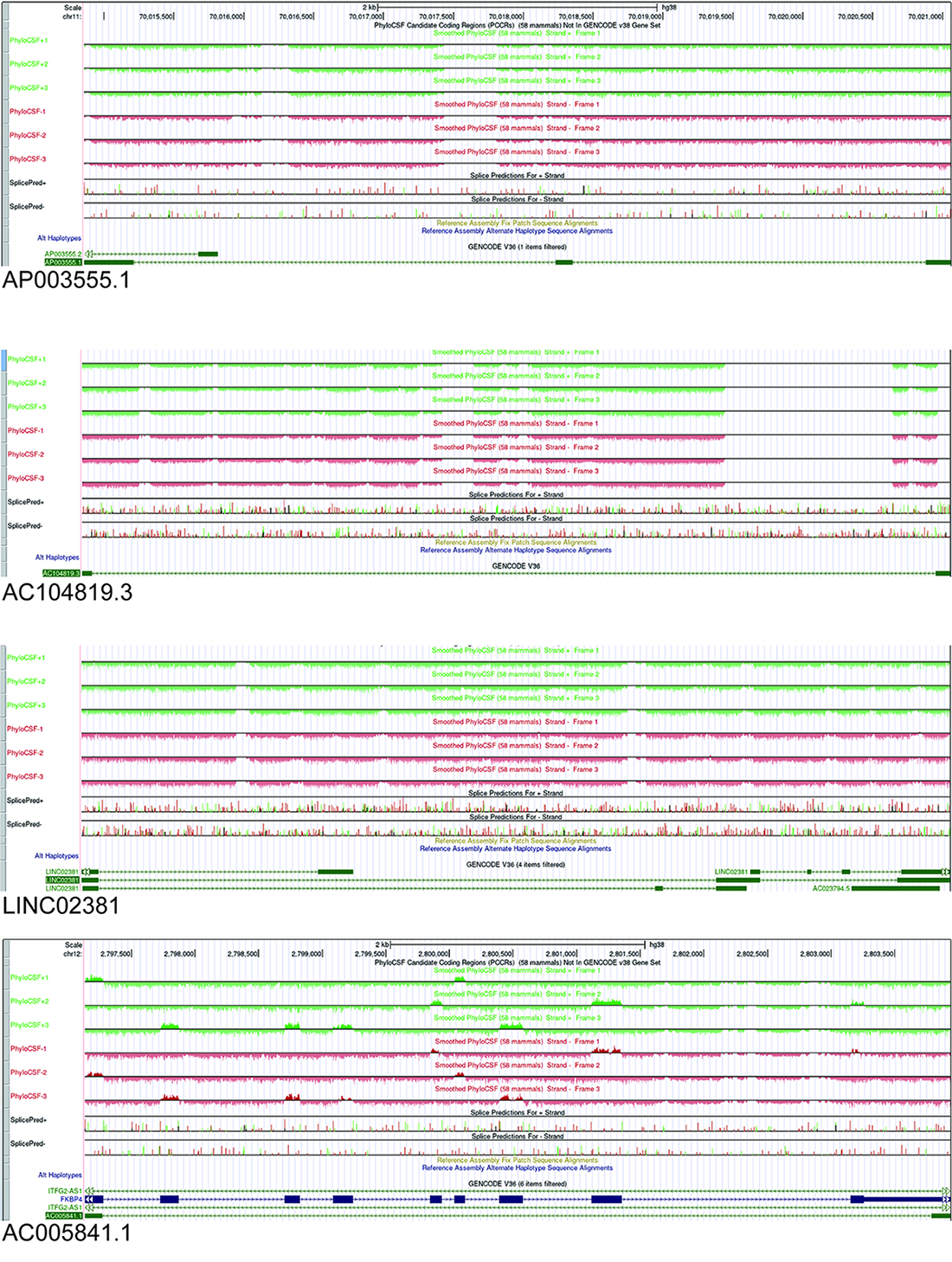

Supplement: Supplementary Figure 2 — The results of 4 FRLs in PhyloCSF. [file Image_2.tif]
